# Urine leukemia inhibitory factor predicts kidney function recovery in patients with acute interstitial nephritis

**DOI:** 10.1172/jci.insight.200389

**Published:** 2026-09-22

**Authors:** Long Qian, Megan L. Baker, Laura Aponte Becerra, Cathleen Liang, Emma Koval, Kyra Shelton, Daris Javed, Gilbert Moeckel, Deepika Kumar, Avi Z. Rosenberg, Michael Kuperman, Tinyi Chu, Wassim Obeid, Xuefei Tian, Chirag R. Parikh, Leyuan Xu, Jonathan Barasch, Shuta Ishibe, Lloyd G. Cantley, Dennis G. Moledina

**Affiliations:** 1 Section of Nephrology and; 2 Department of Pathology, Yale University, New Haven, Connecticut, USA.; 3 Department of Pathology, Johns Hopkins University, Baltimore, Maryland, USA.; 4 Department of Pathology, Cleveland Clinic, Cleveland, Ohio, USA.; 5 Department of Genome Sciences, University of Virginia, Charlottesville, Virginia, USA.; 6 Division of Nephrology, Johns Hopkins University, Baltimore, Maryland, USA.; 7 Division of Nephrology, Columbia University, New York, New York, USA.

**Keywords:** Clinical Research, Nephrology, Biomarkers, Fibrosis, Proteomics

## Abstract

A urine protein called LIF predicts kidney recovery after a common kidney injury called acute interstitial nephritis, potentially guiding treatment decisions and prognosis.

**To the Editor:** Acute interstitial nephritis (AIN) is a common cause of acute kidney injury and can lead to adverse long-term outcomes (1). Histological interstitial fibrosis and tubular atrophy is associated with prognosis but is invasive and subject to interobserver variation (2). Objective, noninvasive biomarkers are needed to improve prognostication. Furthermore, insights into biological pathways associated with recovery may reveal novel treatment targets. Here, we identified and validated urinary leukemia inhibitory factor (LIF) as a prognostic biomarker in AIN using large-scale urinary proteomics and determined its cellular sources and targets.

We measured 5,416 proteins in urine samples collected at biopsy from 55 participants with biopsy-proven AIN, of which 40 achieved renal recovery at 3 months after biopsy (Supplemental Figure 1 and Supplemental Methods; supplemental material available online with this article; https://doi.org/10.1172/jci.insight.200389DS1). LIF was consistently higher in those with renal recovery in both primary (Figure 1A) and sensitivity analyses (Supplemental Figure 2, A–F). Among histological features, LIF was associated with lower interstitial fibrosis and tubular atrophy (Supplemental Table 1). The association of LIF with recovery was consistent regardless of corticosteroid treatment (interaction *P* = 0.63). LIF demonstrated strong discriminatory performance for recovery (AUC, 0.86 [95% CI, 0.76–0.96]) as compared with clinical variables (age, sex, baseline estimated glomerular filtration rate (AUC, 0.67 [95% CI, 0.51–0.83]), resulting in a combined AUC of 0.89 (95% CI, 0.81–0.97) (Figure 1B). Each doubling of LIF was associated with 4.8-fold higher odds of recovery (95% CI, 1.69–13.59) in fully adjusted analysis (Supplemental Table 2). In an independent validation cohort, LIF was associated with recovery (AUC, 0.84 [95% CI, 0.65–1.00]), and adding LIF to a model containing clinical variables improved AUC from 0.88 (95% CI, 0.72–1.00) to 0.97 (95% CI, 0.90–1.00) (likelihood ratio test, *P* = 0.01) (Figure 1C). We performed a sandwich immunoassay measuring urinary LIF, which correlated with proteomics results (R^2^ = 0.89, Supplemental Figure 3B) and showed consistent association with recovery after AIN (Figure 1D and Supplemental Figure 3, C and D). In contrast to AIN, LIF was not associated with recovery after acute tubular injury in discovery (*N* = 19, AUC = 0.58, *P* = 0.68) or validation (*N* = 41, AUC = 0.54, *P* = 0.73) cohorts. There was no difference in urinary LIF between acute tubular injury and AIN in either cohort (test, *P* = 0.64; validation, *P* = 0.37). These observations suggest that LIF is uniquely associated with recovery in AIN. In pathway analysis using urine proteomic findings, the top dysregulated pathways associated with recovery were related to immune responses, such as cytokine storm signaling, macrophage classical activation signaling, and IL-10 and IL-17 signaling (Supplemental Figure 2G).

Next, we used Xenium spatial transcriptomics (10x Genomics) to identify the cellular sources of LIF and its receptor LIFR, potential LIF-mediated cell-cell interactions, and expression of genes downstream of LIFR activation. In AIN biopsies (*N* = 8), mast cells — nearly absent in healthy kidney (*N* = 5) — were the highest LIF expressors (Figure 1E). Distal tubular cells also showed elevated LIF expression in AIN: distal convoluted tubule had 12.1-fold higher expression (*P* = 0.0002); collecting duct had 7.2-fold (*P* = 0.0006); connecting tubule had 6.6-fold (*P* = 0.003); and thick ascending limb had 6.7-fold (*P* = 1 × 10^–6^). Meanwhile, LIFR, expressed by injured proximal tubule cells, endothelial cells, collecting duct cells, and fibroblasts (Supplemental Figure 3A), was reduced in AIN compared with healthy kidneys (*P* < 10^–30^). To determine if increased LIF-LIFR activation occurs despite the low LIFR expression, we examined downstream signaling factors. LIFR stimulation activates SOCS, JAK1/STAT3, PI3K/AKT, and MAPK pathways (Supplemental Figure 3E) (3). SOCS3, in particular, causes degradation of the LIFR complex, suppresses JAK1/STAT3 proinflammatory activation, and promotes cell survival (4). In AIN, cells near high-LIF-expressing cells had higher expression of all of these signaling factors, including marked upregulation of SOCS3 (Figure 1F). Moreover, among injured proximal tubule cells — the most abundant LIFR-expressing cell type — SOCS3 expression correlated with proximity to LIF-expressing cells (Figure 1G). Finally, we used immunofluorescence to confirm protein-level presence of key LIFR downstream signaling factors. Compared with patients with low urinary LIF and nonrecovery, or healthy controls, kidney biopsies from patients with high urinary LIF and recovery showed higher SOCS3 expression and higher phospho-STAT3 nuclear translocation (Figure 1, H and I, and Supplemental Figure 3F), suggesting tissue-level LIFR downstream signaling.

In conclusion, we identified urine LIF as a potential biomarker for kidney recovery in AIN in discovery and validation cohorts and showed LIF expression by mast cells and distal tubular epithelia and activation of LIF downstream pathways in cells spatially proximal to LIF expression. LIF expression directs mesenchymal-to-epithelial transition and tubular repair after experimental acute kidney injury (3). Mast cells can protect against epithelial-mesenchymal transition and fibrosis in murine models (5). Our findings suggest that LIF upregulation may be a response to inflammatory injury and, acting through pathways such as SOCS3, suppress inflammation and promote repair. Strengths of our study include large-scale urinary proteomics, independent validation, and integration with spatial experiments to support mechanistic insights. Limitations include the small sample size, geographic homogeneity, the subjective classification of recovery (though this was addressed through sensitivity analyses), lack of temporal biomarker data, and exploration of only one of several candidate proteins identified on proteomics. Overall, urine LIF holds promise as a prognostic biomarker in AIN, and LIF-related pathways warrant further investigation as therapeutic targets.

For detailed methods, information regarding sex as a biological variable, statistics, study approval, data availability, author contributions, and acknowledgments, see the supplemental materials.

## Conflict of interest

DGM and CRP are coinventors on a pending patent titled “Methods and Systems for Diagnosis of Acute Interstitial Nephritis.” They are also cofounders of Predict AIN, LLC, a diagnostics company that has entered into a technology license and development agreement with Machaon Inc., another diagnostics firm. DGM serves as a consultant for Biohaven Inc. DGM serves as an associate editor for journals *Kidney360* and *Evidence to Action*.

## Funding support

This work is the result of NIH funding, in whole or in part, and is subject to the NIH Public Access Policy. Through acceptance of this federal funding, the NIH has been given a right to make the work publicly available in PubMed Central.

- NIH National Institute of Diabetes and Digestive and Kidney Diseases (NIDDK) Award R01DK128087 to DGM.
- NIH NIDDK Award R01DK126815 to LGC.

## Supplementary Material

Supplemental data

Supporting data values

## Figures and Tables

**Figure 1:**
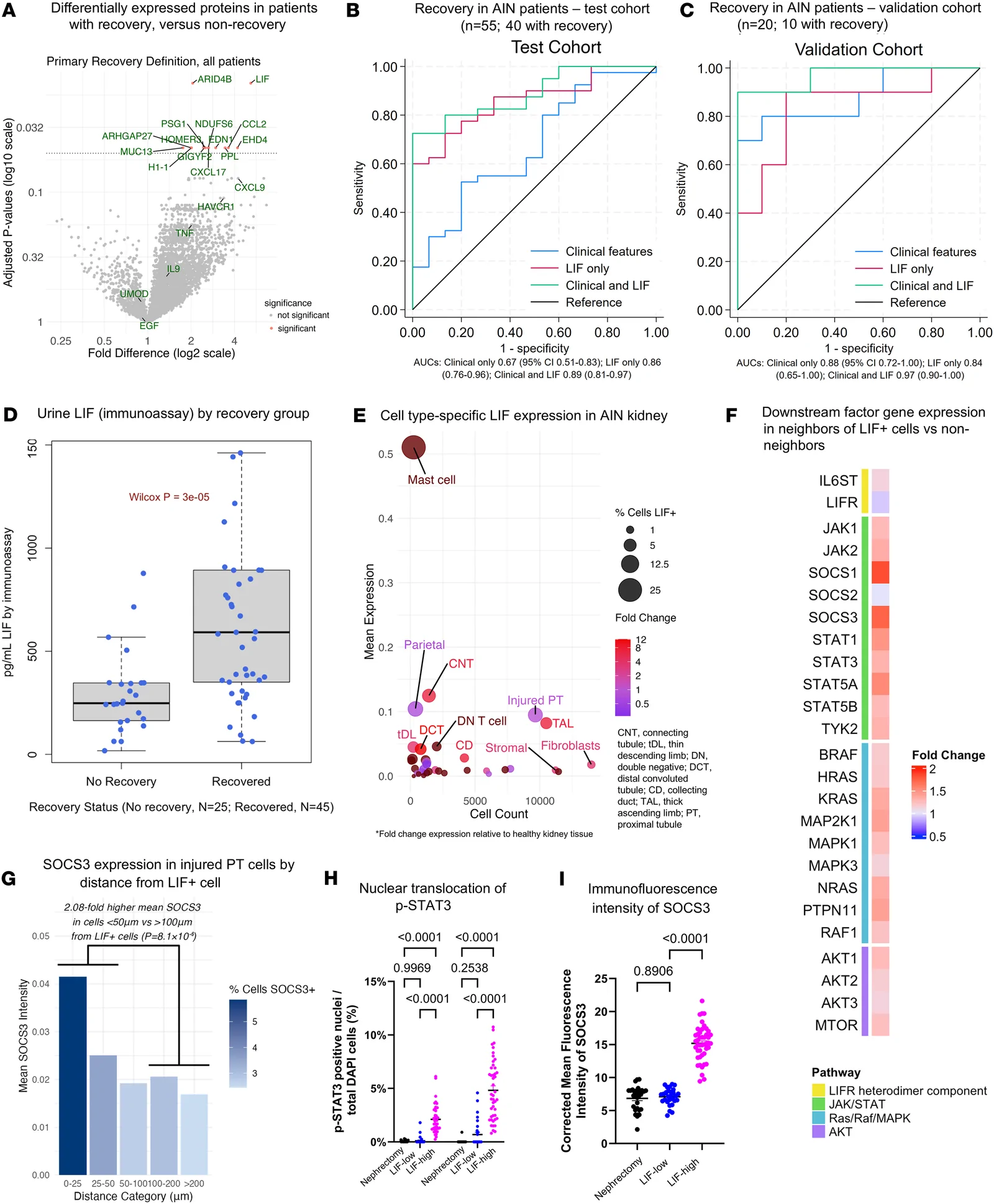
Urinary LIF is associated with recovery after AIN. (**A**) Volcano plot of proteins associated with recovery, controlled for age, sex, and urine creatinine (2-tailed *t* test with multiple testing correction). (**B** and **C**) Predictive models for recovery in AIN in test (**B**) and validation (**C**) cohorts (ROC analysis). (**D**) LIF measured by immunoassay. (**E**) Cells expressing LIF. (**F**) Factors downstream of LIFR signaling, including SOCS3, have higher expression in neighbors of LIF^+^ cells compared with nonneighbors. (**G**) Among injured proximal tubule cells, SOCS3 expression is higher when closer to LIF^+^ cells (Wilcoxon rank-sum test). (**H** and **I**) Immunofluorescence quantification of percentage of p-STAT3^+^ nuclei (within tubular and interstitial compartments) and SOCS3 expression. Fluorescence intensity in arbitrary units, mean ± SEM (2-way ANOVA [**H**] and 1-way ANOVA [**I**]). Two nephrectomy healthy controls (regions of interest [ROI] = 15), 2 AIN low-urine-LIF patients with nonrecovery (ROI = 15), and 3 AIN high-urine-LIF patients with recovery (ROI = 30).

## Version 1 09/22/2026

Electronic publication
