# Supplementary material for: Urine leukemia inhibitory factor predicts kidney function recovery in patients with acute interstitial nephritis
Source: JCI Insight. 2026 Sep 22;11(18):e200389. doi: 10.1172/jci.insight.200389 (PMC13596714; doi:10.1172/jci.insight.200389)
Supplement: Supplementary file 1 [file jciinsight-11-200389-s006.pdf]

Supplemental Materials

Qian L, et al, JCI Insight 2026

Table of contents:

Page	Item
1	Supplemental Figure 1
2	Supplemental Figure 2
3	Supplemental Figure 3
4	Supplemental Table 1
5	Supplemental Table 2
6	Supplemental Methods

Supplemental Figure 1. Creatinine trend of recovery versus non-recovery patients (A) using the primary definition, (B) with borderline recovery cases re-classified as non-recovery, (C) with borderline non-recovery cases re-classified as recovery, (D) with all borderline cases excluded, (E) restricted to only patients with AKI, and (F) restricted to only stage 2 or higher AKI.

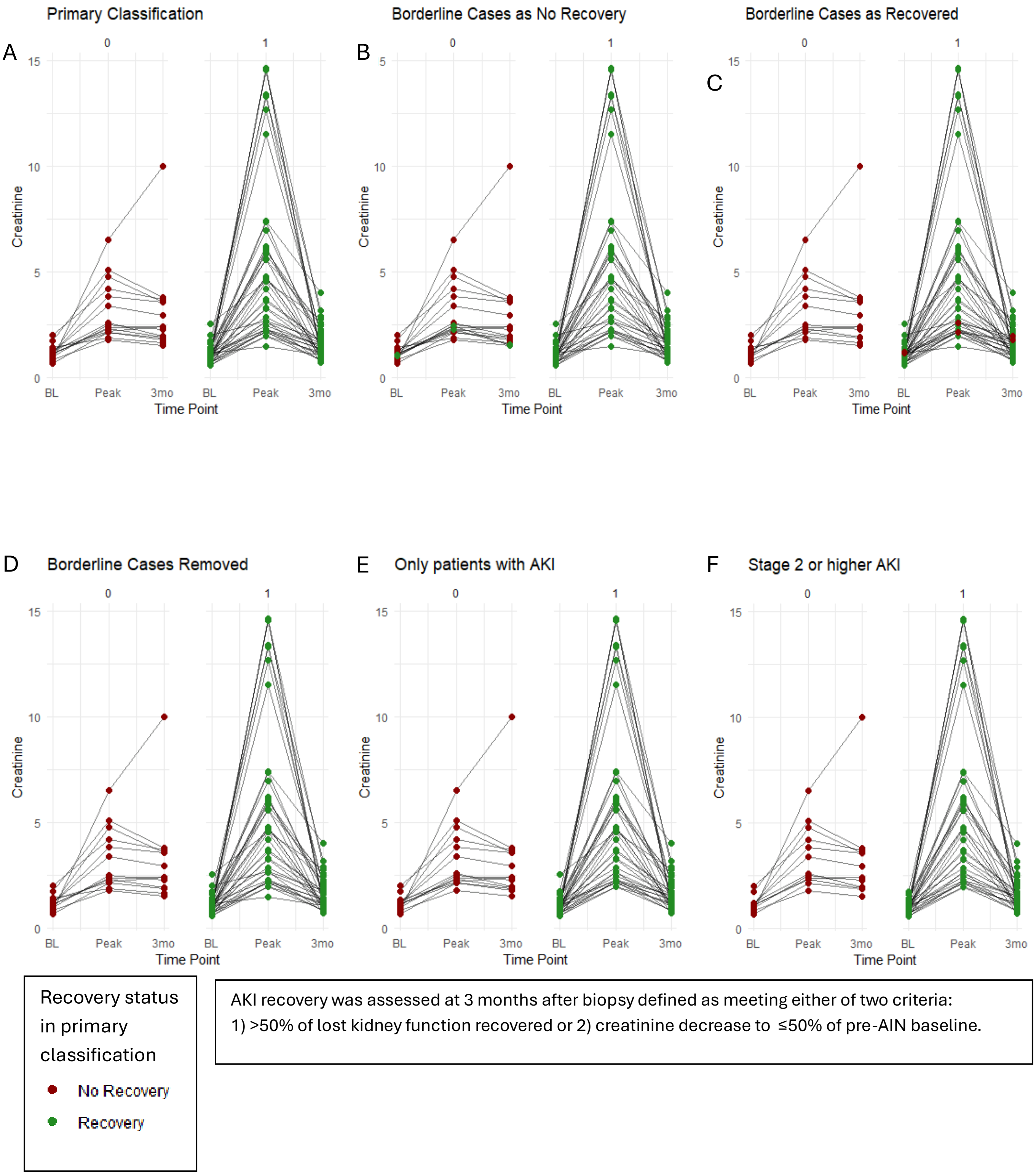

AKI recovery was assessed at 3 months after biopsy defined as meeting either of two criteria:
1) >50% of lost kidney function recovered or 2) creatinine decrease to ≤50% of pre-AIN baseline.

Supplemental Figure 2. (A-F) Sensitivity analyses of differentially expressed proteins between recovery and non-recovery groups, with borderline recovery cases re-classified as non-recovery (A), as recovery (B), or excluded (C), with analysis restricted to only patients with AKI (D), only stage 2 or higher AKI (D), and using the primary definition with additional adjustment for albuminuria (F). (G) Pathways identified from the differentially expressed proteins in recovery versus non-recovery in AIN patients with stage 2 or higher AKI*; arrows indicate pathways that include LIF. *Pathway analysis done using the 103 proteins identified from patients with stage 2 or higher AKI as this was the most comprehensive set of proteins.

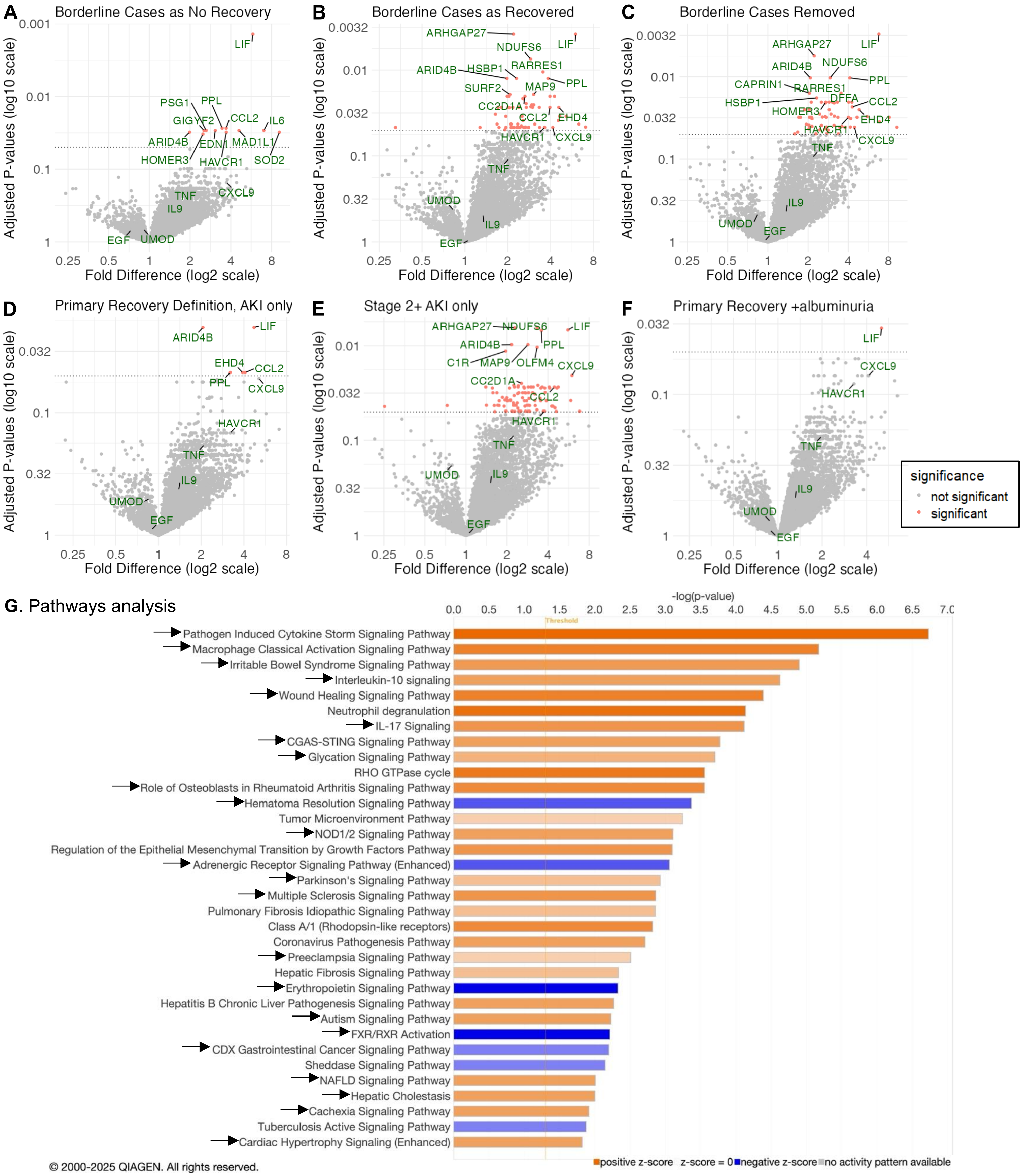

Supplemental Figure 3. (A) Cell-type specific LIFR expression in AIN. PT, proximal tubule; EC, endothelial cell; CD, collecting duct; CNT, connecting tubule; tDL, thin descending limb; TAL, thick ascending limb in Xenium spatial transcriptomic analysis. (B) LIF levels measured using direct immunoassay correlate closely with proteomic analysis. (C) Immunoassay LIF:Creatinine ratio is higher among patients who recovered after AIN. (D) Immunoassay-measured LIF and LIF:Cr ratio both predict recovery among the combined test and validation cohorts. (E) Schematic diagram of LIFR downstream signaling pathways and factors. (F) Representative immunofluorescence images of human kidney biopsy sections from nephrectomy healthy controls, AIN patients with low-urine-LIF/non-recovery, and AIN patients with high-urine-LIF/recovery. Sections were stained for p-STAT3 (Tyr705) (red), SOCS3 (green), and DAPI (blue).

A. Cell-type specific expression of LIFR in AIN

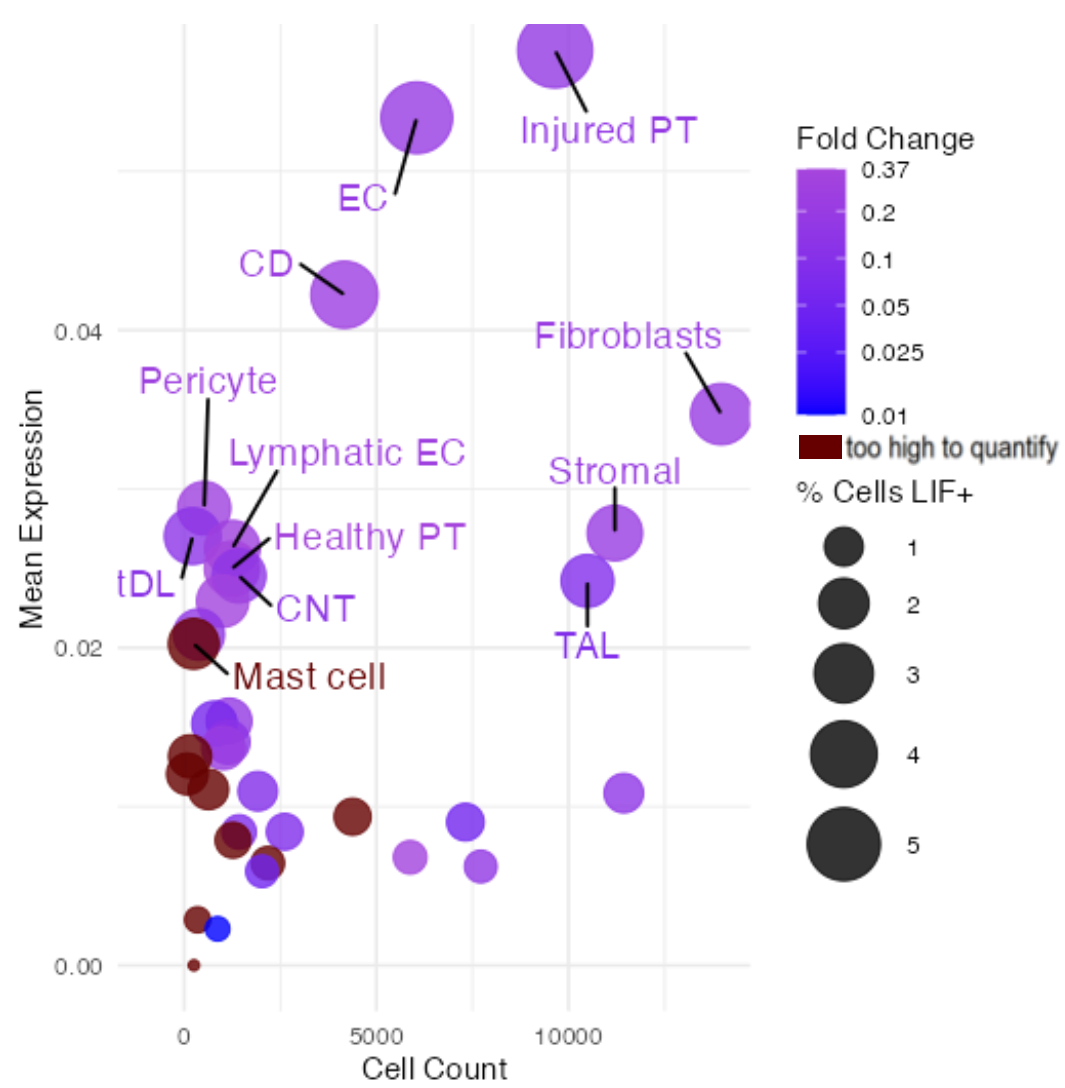

B. Correlation between immunoassay and proteomic LIF levels

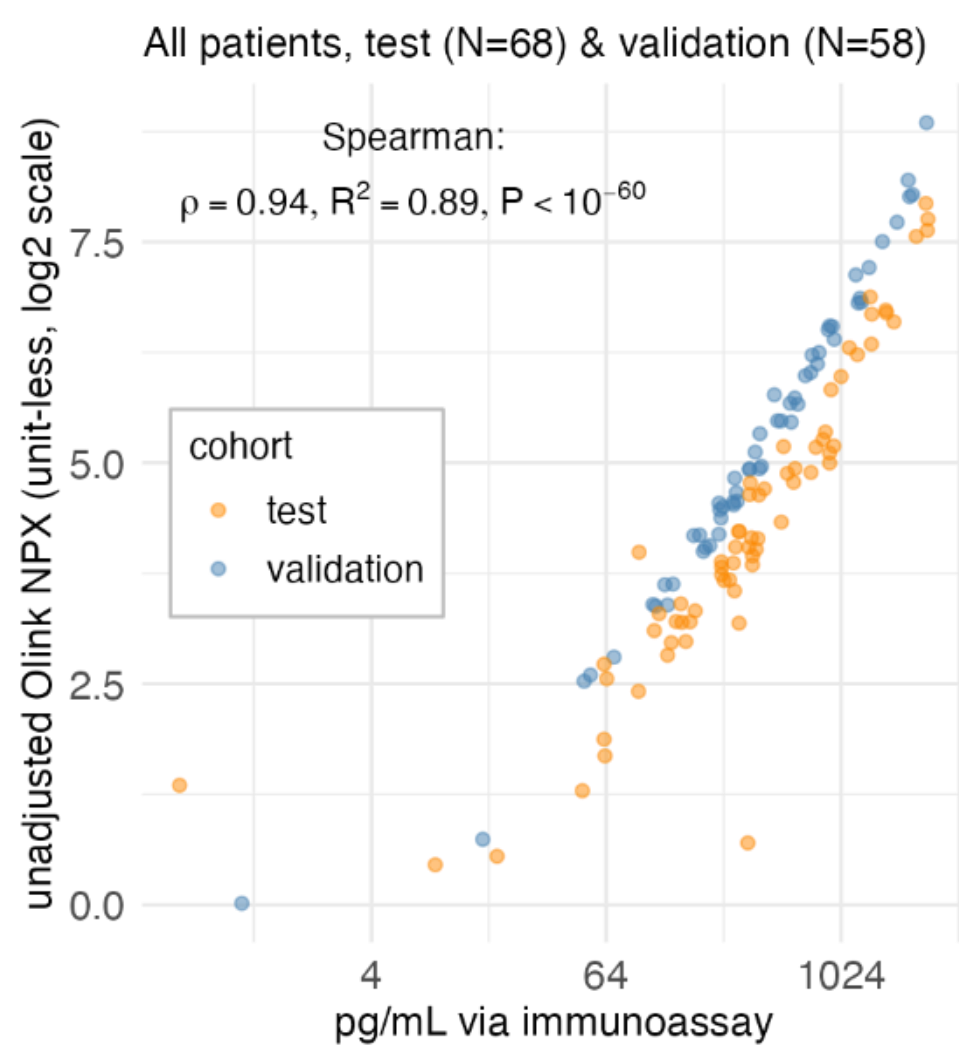

C. LIF:creatinine ratio (pg/mg) by recovery among AIN patients

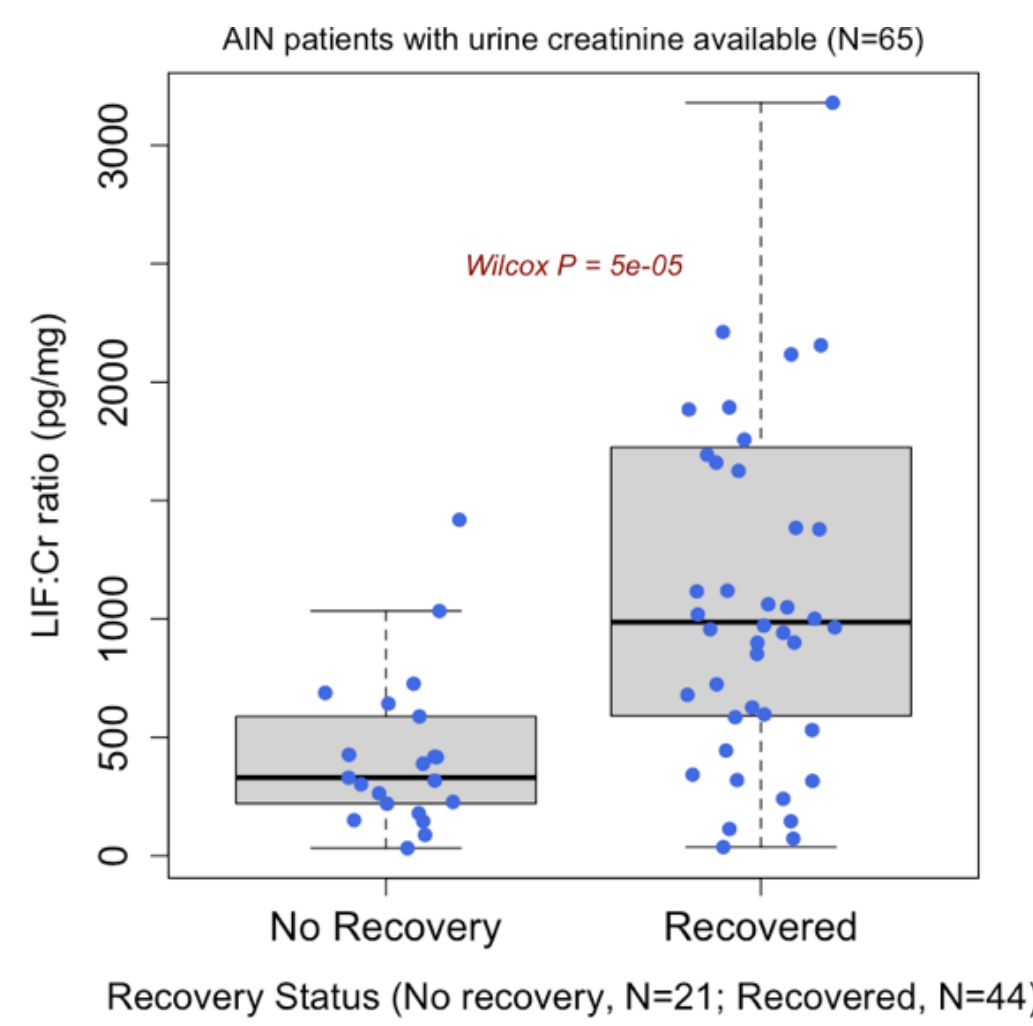

D. Immunoassay-measured LIF and LIF:Creatinine ratio predicts recovery

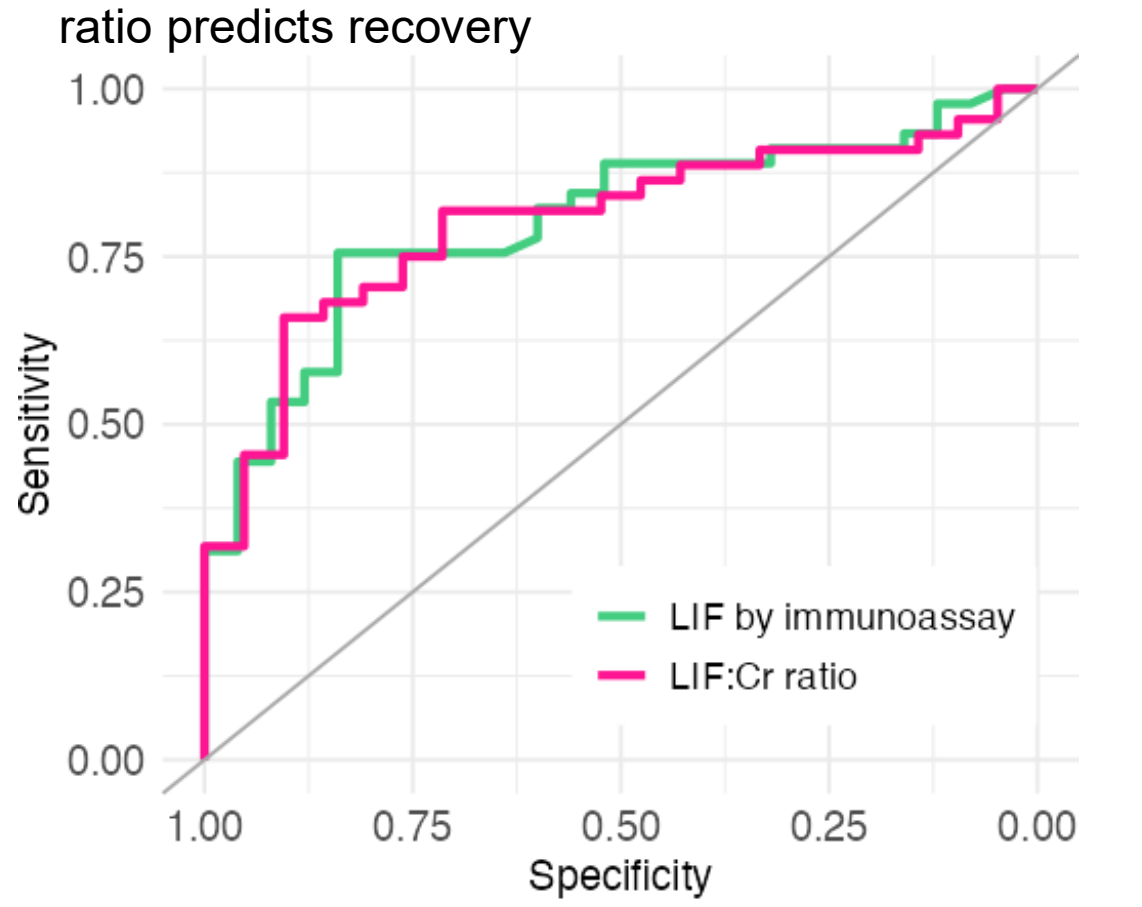

AUCs: LIF immunoassay, 0.80 (95% CI 0.70-0.91); LIF:Cr ratio, 0.80 (0.69-0.91)

E. LIFR downstream signaling factors

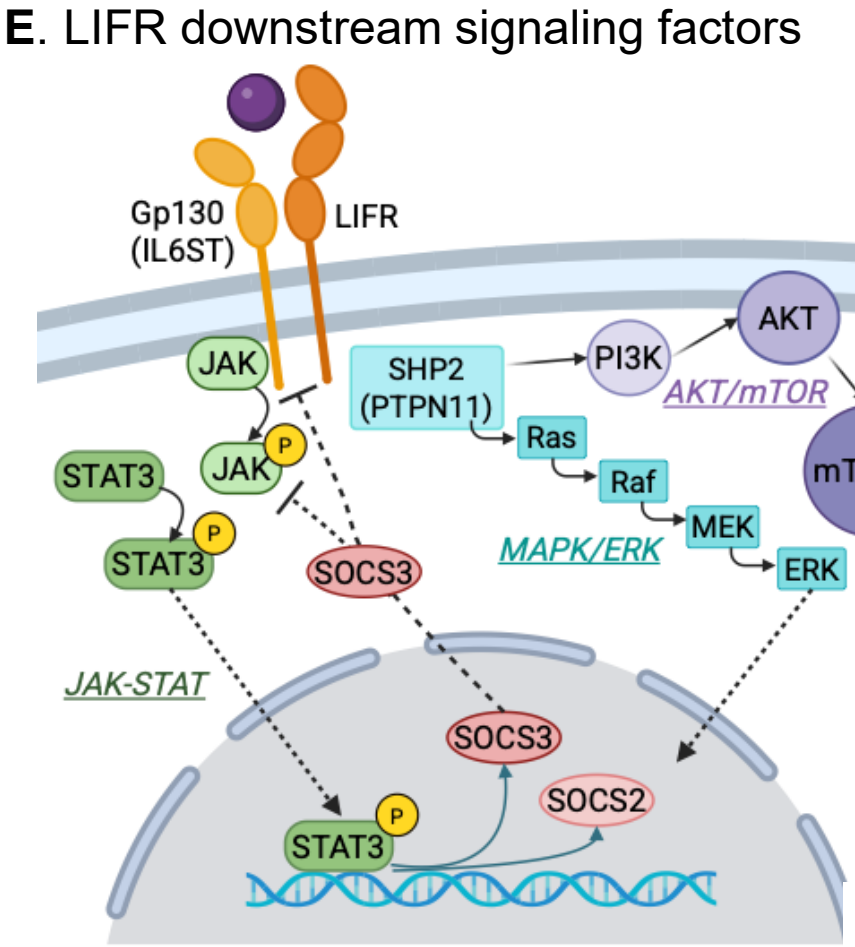

Image created in BioRender

F. Representative images of SOCS3 and pSTAT3 dual immunofluorescence

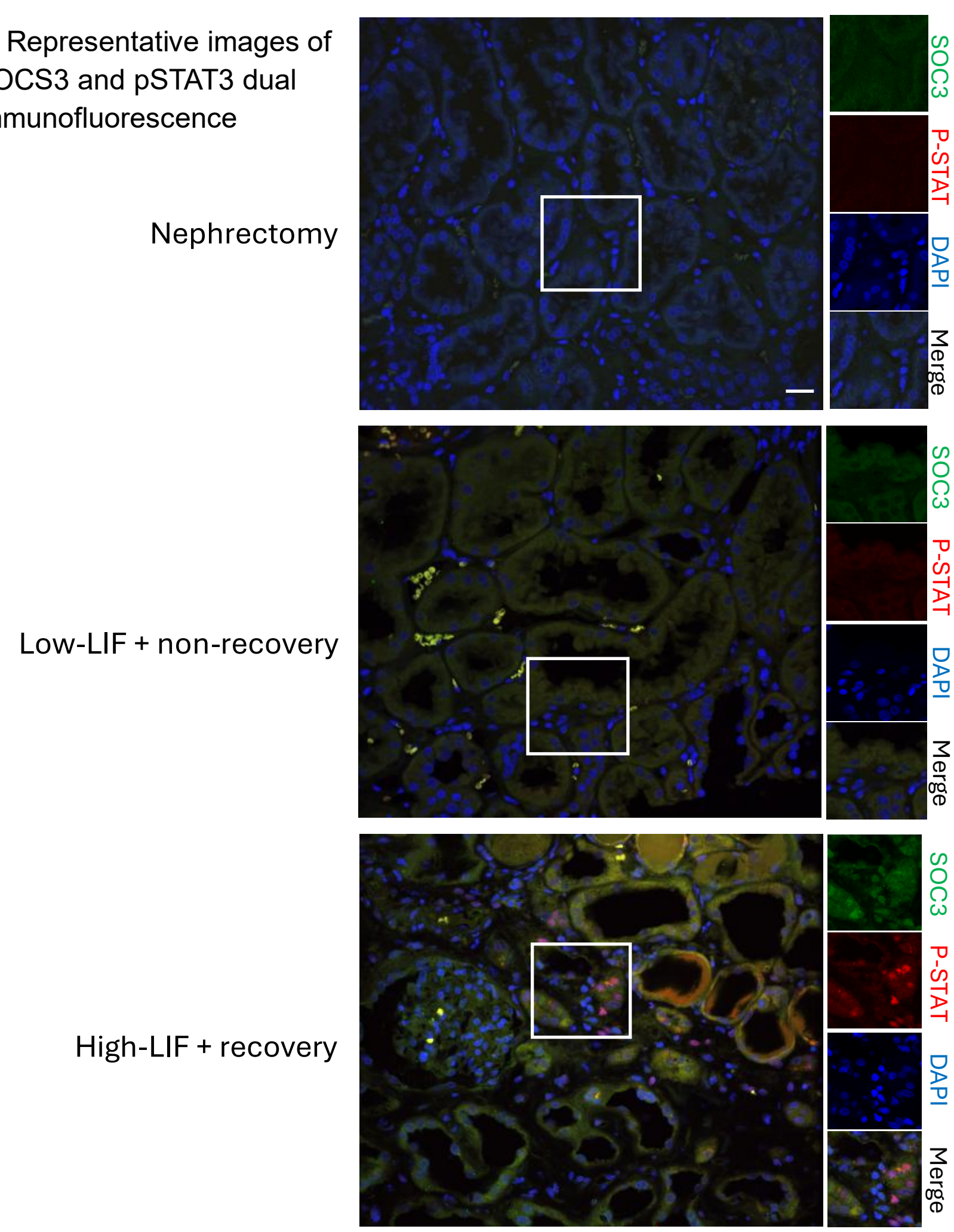

Supplemental Table 1. Among 4 proteins consistently identified across all sensitivity analyses, LIF and CCL2 were associated with higher IFTA grade (A);

Histopathological feature	Protein	P-value	Estimate
**Tubulitis (presence or absence)**	LIF	0.527	0.450
	ARID4B	0.280	0.405
	PPL	0.282	0.609
	CCL2	0.855	0.104
**IFTA (% of cortex)**	LIF	0.009	-0.044
	ARID4B	0.223	-0.010
	PPL	0.299	-0.014
	CCL2	0.031	-0.028
**Inflammatory infiltrate (% of non-scarred cortex)**	LIF	0.608	-0.255
	ARID4B	0.631	0.120
	PPL	0.950	0.025
	CCL2	0.747	-0.127
**Glomerulosclerosis (% of total glomeruli that were sclerlosed)**	LIF	0.484	-0.009
	ARID4B	0.466	-0.005
	PPL	0.932	0.001
	CCL2	0.408	-0.009
**ATI (severity graded 1-4)**	LIF	0.504	0.330
	ARID4B	0.917	0.026
	PPL	0.686	-0.158
	CCL2	0.245	0.451

Supplemental Table 2. Sequential nested models predicting recovery, using LIF as measured by proteomic experiment (NPX), or by direct immunoassay

Main Predictor	Model*	Odds ratio (95% CI)	P-value
LIF (NPX), per doubling	1	3.50 (1.58-7.75)	0.002
LIF (NPX), per doubling	2	4.75 (1.72-13.18)	0.003
LIF (NPX), per doubling	3	4.80 (1.69-13.59)	0.003
LIF (immunoassay), per 100 mg/mL increase	1	1.45 (1.14-1.86)	0.003
LIF (immunoassay), per 100 mg/mL increase	2	1.81 (1.11-2.96)	0.02
LIF (immunoassay), per 100 mg/mL increase	3	1.83 (1.09-3.06)	0.02
LIF:Cr ratio only, per 100 pg/mg increase	1	1.28 (1.09-1.49)	0.002
LIF:Cr ratio only, per 100 pg/mg increase	2	1.40 (1.08-1.82)	0.01
LIF:Cr ratio only, per 100 pg/mg increase	3	1.39 (1.07-1.80)	0.01

*Model 1 includes the main predictor only.
*Model 2 includes the main predictor as well as age at biopsy, sex, and baseline eGFR.
*Model 3 includes the main predictor, all covariates of Model 2, and additionally histological IFTA grade.

Supplemental methods

Participants and setting:

The discovery cohort included participants enrolled in the Yale biopsy cohort, which has been previously described.[1] Briefly, we prospectively enrolled patients undergoing a kidney biopsy at 2 Yale-affiliated sites in New Haven, Connecticut, USA: Yale New Haven Hospital and Saint Raphael's Hospital. Patients in this study were enrolled between September 2020 and November 2024. We excluded patients undergoing biopsies for evaluation of transplanted kidneys or kidney malignancies, participants who failed to provide a urine sample for analysis, and participants who had insufficient tissue for histological diagnosis.

The validation cohort was also comprised of participants from the Yale biopsy cohort, enrolled at earlier dates (January 2015 - June 2018) and included in a previously published study.[2]

Study approval:

This study was approved by Yale institutional review board (approval numbers 2000027890 and 11110009286). All participants provided written, informed consent.

AIN definition:

In this analysis, we included only patients with AIN, adjudicated as follows by either two of three pathologists or two nephrologists. Three pathologists independently reviewed samples and provided histological diagnoses; if at least two of the three gave AIN as the first diagnosis, then the patient was deemed as having AIN. Two nephrologists independently reviewed patients' biopsy reports as well as clinical data in the electronic health record including post-biopsy clinical diagnoses, treatment plans, and outcomes, and after disagreements were resolved by in-person discussion, patients were included in the study if the final agreed-upon diagnosis was AIN. Diagnosis of AIN was not to the exclusion of other possible concurrent but deemed unrelated diagnoses such as acute tubular injury or diabetic nephropathy.

Outcome definition:

The primary outcome of recovery was defined as meeting either of the two criteria below:

A) Having AKI defined by KDIGO criteria (peak creatinine ≥1.5x baseline),[3] AND at 3 months after biopsy, having creatinine less than or equal to 1.5x baseline creatinine, calculated as:
3-month creatinine ÷ baseline creatinine < 1.5

B) At 3 months after biopsy, having recovered at least 50% of the increase in creatinine from baseline, calculated as:
(peak creatinine – 3-month creatinine) ÷ (peak creatinine – baseline creatinine) > 0.5

The same criteria were applied to define recovery in the validation cohort except that 6-month creatinine was used rather than 3-month creatinine due to data availability.

Sensitivity analyses: Given the subjective nature of defining recovery thresholds, we conducted sensitivity analyses. "Borderline" cases were defined as a having criterion A ratio (3-month creatinine ÷ baseline creatinine) between 1.3 and 1.7, and a criterion B value ((peak creatinine – 3-month creatinine) ÷ (peak creatinine – baseline creatinine)) between 0.3 and 0.7. There were 4 patients in this category, 2 initially classified as recovered and 2 initially classified as non-recovery. For sensitivity analyses, we repeated the main analyses after reclassifying all four as non-recovery, then all as recovery, and finally by excluding them entirely (N=51). We also conducted sensitivity analyses restricted to patients with at least stage 1 AKI, defined as peak creatinine ≥1.5x baseline (N=52), and separately for those at least stage 2 AKI, defined as peak creatinine ≥2x baseline (N=48).

Urine proteomics:

Urine samples were collected at the time of clinically indicated biopsy. Samples were transported on ice, centrifuged at 2000 RPM for 12 minutes at 4°C, the supernatant aliquoted into 2mL volumes, and then stored at -80°C. The sample processing protocol and biorepository tracking details are described in a prior publication.[4] 75uL of each urine sample was sent to Olink, and the proteomic experiment was performed after a single controlled thaw.

Urine proteomic measurements were performed by Olink Proteomics using a commercially available, manufacturer-validated panel named ExploreHT. The Olink Proximity Extension Assay (PEA) is a high-throughput, multiplexed, proteomic platform. Two PEA probes (oligonucleotide-labeled monoclonal or polyclonal antibodies) separately bind each target protein to minimize cross-reactivity. Upon binding, the complementary probes for each target hybridize and extend, generating a unique sequence used for identification of each specific protein. The amounts of known sequences are translated into Normalized Protein eXpression (NPX) units on a log2 scale derived from count values. Olink performed measurements blinded to case status and provided results to investigators. Olink had no role in statistical analysis or publication.

Quality control: Olink thresholds for quality control are internally set such that samples or assays are flagged as "Fail" if the total counts, or several control counts, fall below minimum count thresholds, and “Fail” samples or assays are recommended for exclusion. Those flagged as "Warn" meet a higher threshold and are recommended for cautious interpretation rather than automatic exclusion. In our dataset, no proteins received a "Fail" flag. One protein, ApoE, was flagged as "Warn" in all samples and was excluded from analysis. An additional 67 proteins were flagged as "Warn" in 5 samples (4 AIN, 1 ATI). These protein-sample combinations were excluded, while the other proteins in the 5 samples and the same proteins in other samples were retained. The 67 proteins were:

AFM, APCS, APOA1, APOA4, CFHR2, APOD, CFHR4, CFI, CLU, ATRN, CFP, PZP, CPB2, B2M, RBP4, F12, ITIH1, A1BG, ECM1, C4B, BTD, C1S, SELENOP, F13B, ITIH4, HABP2, C5, LGALS3BP, C1RL, C3, SERPINA1, F2, KLKB1, HRG, C7, FGG, LPA, SERPINA3, FGA, LRG1, IGLC2, C8B, FN1, SERPINA7, LUM, SERPINF1, SERPINA4, SERPIND1, CD5L, SERPINC1, LYVE1, SERPINF2, SERPINA5, MBL2, PLG, CFB, SERPING1, SERPINA6, CFD, PGLYRP2, ORM1, PPBP, TTR, VTN, GC, TF, CFH.

Statistical analysis:

Generating adjusted-NPX values for primary analysis: We created a linear regression model with age, sex, and urine creatinine as independent variables and raw NPX values as the dependent variable. Residuals from this model, referred to as adjusted-NPX values, were used for all subsequent analyses. For further adjustment for albuminuria, dipstick proteinuria was added to this same model. Similar methods were previously used by other proteomic studies.[5,6]

Associations with recovery: For each protein, we conducted a two-tailed t-test comparing adjusted-NPX values between recovered and non-recovered patients (as defined above), including reclassifications from sensitivity analyses. Multiple testing correction was performed using the Benjamini-Hochberg (BH) method to obtain q-values, with <0.05 as the threshold for statistical significance.

Histological associations: Histological features were extracted from clinical pathology reports: tubulitis (present/absent), IFTA (percent of cortical involvement), glomerulosclerosis (percent of glomeruli sclerosed), and ATI (graded 1-4, with 4 being most severe). For each feature, linear regression was performed with the histological variable as the independent variable and protein adjusted-NPX as the dependent variable.

Urine Immunoassay

Urine from the same patients included in the Olink experiment (75uL aliquot) was used to measure LIF using the Quantikine ELISA Kit (Cat: DLF00B) from R&D (Minneapolis, MN, USA). This experiment was performed in Baltimore, MA, USA. All other experiments and all analyses done in New Haven, CT, USA.

Xenium spatial transcriptomics analysis:

***Dataset and Cell Type Classification*:** We analyzed LIF and LIFR expression patterns using a Xenium spatial transcriptomics dataset containing 730,011 cells from 35 patients across 4 disease categories (AIN: 128,823 cells, 8 patients; Reference: 80,593 cells, 5 patients). Spatial transcriptomics data were processed using Seurat v5, with quality control removing cells with zero UMIs. After normalization and variable feature identification, we integrated tissue sections using Harmony to correct batch effects while preserving biological heterogeneity. Following PCA (30 components), we performed Leiden clustering at multiple resolutions (0.4-1.2), selecting resolution 0.4 based on optimal silhouette scores. Clusters were annotated through differential gene expression analysis and spatial mapping, yielding 27 core cell types. Major immune populations (T cells, macrophages) underwent supervised sub-clustering using lineage-defining markers (e.g., CD4, FOXP3, CCR7) to identify detailed subtypes. UMAP visualization was performed on the integrated embedding for dimensional reduction. For initial expression analysis, we combined cortex and medulla tissues within each disease category (AIN_C + AIN_M = AIN; REF_C + REF_M = REF; ATI_C + ATI_M = ATI) to maximize statistical power, then performed cortex-specific analyses for both expression comparisons and spatial signaling studies to avoid the possibility of bias by region-specific effects and differences in region representation between tissue types.

Expression Quantification: Expression statistics were calculated using normalized count data from the Seurat object's "data" layer. For each cell type and gene combination, we computed: (1) **mean expression per cell** by averaging normalized expression values across all cells of that type, followed by cell type-specific fold-change between AIN and reference, and (2) **percentage expressing** as the proportion of cells with detectable expression (>0). Cell type-specific fold-change of expression was not computed (“Unable to quantify” in Figure 1 and Supplemental Figure 3) for cell types with undetectable mean expression level in reference or AIN tissue. We performed pairwise statistical comparisons comparing AIN vs REF, assessing **mean expression differences** using Wilcoxon rank-sum tests. P-values were adjusted for multiple testing using the Benjamini-Hochberg false discovery rate (FDR) correction applied separately for each gene (LIF and LIFR), with significance set at FDR < 0.05.

Spatial Signaling Analysis: To infer sites of LIF-LIFR communication potential, we identified and quantified spatially co-localized LIF+ and LIFR+ cell pairs within cortex tissues. We calculated Euclidean distances between all LIF+ and LIFR+ cell pairs within each tissue. Signaling pairs were defined as LIF+ cells located within 20μm of LIFR+ cells, representing a conservative distance threshold where paracrine signaling is highly probable. Autocrine interactions (same cell expressing both genes) were excluded. Signaling density was calculated as total signaling pairs per tissue area (pairs/mm²), using previously measured whole patient tissue areas to account for differences in tissue sampling between conditions.

Immunofluorescence

Staining. Formalin-fixed paraffin-embedded (FFPE) sections (4 μm) from 2 nephrectomy samples, 3 AIN patients with high urine LIF who recovered, and 2 AIN patients with low urine LIF and non-recovery were included. Sections were deparaffinized in xylene and rehydrated through a graded ethanol series, as previously described.[7] Antigen retrieval was performed by heating the sections in Tris-EDTA buffer (10 mM Tris, 1 mM EDTA, 0.05% Tween-20, pH 9.0) for 10 minutes. Following permeabilization with 0.2% Triton X-100 in PBS for 10 minutes, sections were blocked using 3% BSA and 0.1% Triton X-100 in PBS for 1 hour at room temperature. The slides were then incubated overnight at 4°C with the following primary antibodies: rabbit anti-phospho-STAT3 (Tyr705) (1:75; Cell Signaling Technology, catalog 9145) and mouse anti-SOCS3 (1:75; Abcam, catalog ab14939). After three washes in PBS,

sections were incubated for 1 hour at room temperature with Alexa Fluor 488-conjugated goat anti-mouse IgG (1:200; Invitrogen, catalog A11029) and Alexa Fluor 594-conjugated donkey anti-rabbit IgG (1:200; Invitrogen, catalog A21207). Slides were mounted using SlowFade Gold Antifade Mountantwith DAPI (Invitrogen, catalog S36938). For negative controls, parallel sections were processed identically, except that primary antibodies were replaced with an equal volume of blocking buffer.

Image Acquisition and Quantification. Images were acquired using an Andor CSU-W1 spinning disk confocal microscope equipped with a Nikon Ti-E CFI Plan Apochromat Lambda 40× oil immersion objective. Image processing and quantitative analyses were performed using NIH ImageJ software. For spatial quantification, the renal tubular and interstitial compartments were delineated based on morphological features using the DAPI counterstain. Nuclear p-STAT3 activation was quantified by calculating the percentage of DAPI-positive nuclei that colocalized with the p-STAT3 signal within each defined compartment. SOCS3 expression was assessed by measuring the mean fluorescence intensity (MFI) per field of view after background subtraction.

Statistics. Data were presented as the mean ± SEM. The percentage of p-STAT3-positive nuclei were compared and P-value determined by two-way ANOVA followed by Šidák's multiple comparisons test. SOCS3 mean fluorescence intensity (MFI, arbitrary units) was compared and P value determined by one-way ANOVA followed by Tukey's post hoc test. Statistical analyses were performed using GraphPad Prism software (version 11.0).

Sex as a biological variable:

Sex was categorized as male or female based on patient self-report.

Conflicts of interest:

DGM and CRP are co-inventors on a pending patent titled "Methods and Systems for Diagnosis of Acute Interstitial Nephritis." They are also co-founders of Predict AIN, LLC, a diagnostics company that has entered into a technology license and development agreement with Machaon, Inc., another diagnostics firm. DGM serves as a consultant for Biohaven, Inc. DGM serves as an associate editor for journals Kidney360 and Evidence to Action.

Funding:

- National Institutes of Health (NIH) National Institute of Diabetes and Digestive and Kidney Diseases Award (NIDDK) R01DK128087, to DGM.
- NIH NIDDK Award R01DK126815, to LGC.

This work is the result of NIH funding, in whole or in part, and is subject to the NIH Public Access Policy. Through acceptance of this federal funding, the NIH has been given a right to make the work publicly available in PubMed Central.

Acknowledgements*:*

We wish to thank the participants of the Yale biopsy study, without whom this study would not have been possible.

Data availability:

Raw data are accessible through the Yale Dataverse database at https://doi.org/10.60600/YU/YCOAZI. Data underlying figure panels are provided in the Supporting Data file.

Author contributions

LQ, MLB, CRP, SI, LGC, and DGM conceived and designed the study.

LQ, MLB, LAB, CL, EK, KS, DJ, XT, and LX conducted experiments and acquired data. WO performed biomarker experiments.

SI, CRP, LGC, and DGM provided supervision.

GM, DK, MK, and AR performed histopathological assessment of kidney tissue.

JB provided scientific guidance.

LQ, MLB, TC, and XT analyzed the data.

LQ and DGM wrote the manuscript, and all authors reviewed, edited, and approved the final version.

Supplemental references

1. Moledina, D.G., *et al.* Urine TNF-alpha and IL-9 for clinical diagnosis of acute interstitial nephritis. *JCI Insight* **4**(2019).
2. Moledina, D.G., *et al.* Identification and validation of urinary CXCL9 as a biomarker for diagnosis of acute interstitial nephritis. *The Journal of Clinical Investigation* **133**(2024).
3. Kidney Disease: Improving Global Outcomes (KDIGO) Acute Kidney Injury Work Group. KDIGO Clinical Practice Guideline for Acute Kidney Injury. . *Kidney inter., Suppl.* **2**, 1-138 (2012).
4. Nadkarni, P.M., Kemp, R. & Parikh, C.R. Leveraging a clinical research information system to assist biospecimen data and workflow management: a hybrid approach. *J Clin Bioinforma* **1**, 22 (2011).
5. Wen, Y., *et al.* Urine Proteomic Signatures of Kidney Function Decline after Hospitalization: TH-PO076. *Journal of the American Society of Nephrology* **35**, 10.1681/ASN.20245v20243jz20244vr (2024).
6. Lopez-Silva, C., *et al.* Circulating Protein and Metabolite Correlates of Histologically Confirmed Diabetic Kidney Disease. *Kidney Med* **6**, 100920 (2024).
7. Tian, X., *et al.* Profilin1 is required for prevention of mitotic catastrophe in murine and human glomerular diseases. *J Clin Invest* **133**(2023).
